# Over-Working

**Published:** 1854-02

**Authors:** 


### OVER-WORKING.

Over effort of body or mind, especially if protracted, often induces incurable forms of disease, and should be avoided as any other cause of sickness and suffering. Young clergymen are particularly liable to this fault, and, before they are aware of it, they discover that a constitution which they believed impregnable is ruined, and the prospect of a long life before them of comparative inertia weighs upon the spirit like an immovable incubus. But the necessity of making a living in some way soon becomes apparent, and one of the first thoughts is to teach school, or keep store, or go on a farm. Some, whose circumstances are easy, conclude hastily to abandon preaching, or wait and see if the injury will not repair itself.

The following letter was addressed by me to a young clergyman of an energetic temperament, who had written to me several times. It may be generally useful. The points touched upon were in answer to suggestions of his own, and may be understood without giving his letter at length. He preached with great animation, "loud, and gesticulated violently, leading in the singing, and this two or three times a day sometimes."

New York, January 26th, 1854.

Dear Sir:—Yours is received. Over-exercise always injures, and if you have done it to "breaking down," then you have done yourself a great wrong, and you must exercise in moderation to repair it; it is much like a burnt finger or a frozen toe, the best repair is heat in a milder form, or cold in a milder form. You are "longing for action:" that is well; then act away, in moderation; work the body moderately, work the mind moderately, and you will almost certainly recuperate. "Working on a farm" will do you many times greater good than "going to the South" to feed and lounge about, and do nothing. I do not object to your working on a farm, but two things are requisite to you, as you have an active, vigorous mind. The work must be pecuniarily remunerative, and it must be connected with some mental labor, such as preaching somewhere, every two or three days; then, while you are at work, you can be studying out your sermons, and perhaps they will be about as good sermons as you have ever made, if not better.

You are "easily worried;" then you are not a philosopher. Take the world easy; you will get through it soon enough. The wood-chopper does most who pulls off his coat and goes at it leisurely, and so will you, in the long run, in cutting down tall sinners. Do not "clerk on, and rant on," and stave away, as if you would drive men into the gospel pen, as butchers do sheep and pigs, by the more noise they make. Imagine yourself a Judge on the supreme bench in Washington, and speak with their dignity and deliberation and weight, especially as they speak for temporal, you for immortal interests.

You have intimated that perhaps it would be better for you to "embark in trade of some kind for a year or two," until your health is fully re-established, and thus "wait." A young man with the world before him, at the threshold of professional life, cannot afford in these stirring times to "wait" for anything; he must force it up, attempt everything with energy and indomitable perseverance. If your health is not fully restored, you will do one of two things, go down from your great work, or settle down to be a common, plodding, sickly preacher. Ought you to do either? With youth, health, and a right heart, why may you not become an eminent and efficient leader in your church? Think of it.

